# Integration of Physiological Analysis and Untargeted Metabolomics to Explore Differences in Quality Among Four Sweet Cherry Cultivars

**DOI:** 10.3390/foods14183207

**Published:** 2025-09-15

**Authors:** Guoqin Li, Xiaosa Yang, Zhonghua Cao, Fei Li, Guifeng Li, Erihemu

**Affiliations:** 1 Laboratory for Food Storage and Processing, School of Food Science, Shanxi Normal University, Taiyuan 030031, China; 2 Shanxi Engineering Research Center of Microbial Application Technologies, School of Food Science, Shanxi Normal University, Taiyuan 030031, China

**Keywords:** sweet cherry, fruit quality, differential metabolites, untargeted metabolomics

## Abstract

This study compared the quality parameters of four popular sweet cherry fruits (“Tieton”, “Pioneer”, “Sunburst”, and “Huangmi”) in Shanxi Province and used untargeted metabolomics to analyze the differential metabolites (DMEs) among them. The results showed that the four fruits have distinct differences in their skin color, texture, size, weight, and solid-to-acid ratio. Notably, “Huangmi” fruit showed greater physical damage and bitterness and lower overall likeability than the other three fruits after short-distance road transportation. Untargeted metabolomics identified 97 DMEs among the four fruits. Specifically, the levels of 44 DMEs (such as cellobionate, allose, L-histidine, kaempferol, ascorbic acid, cinnamaldehyde, and Qing Hau Sau), 22 DMEs (such as raffinose, neochlorogenic acid, epicatechin, carvone, and (S)-norcoclaurine), 9 DMEs (such as melibiitol, 3′-ketolactose, and all-trans-retinoic acid), and 3 DMEs (D-maltose, shikimic acid, and selenocysteine) were highest in the “Huangmi”, “Sunburst”, “Pioneer”, and “Tieton” fruits, respectively. Moreover, the red cultivars (“Tieton”, “Pioneer”, and “Sunburst”) showed a higher citrulline content than the yellow cultivar (“Huangmi”). This study can serve as a reference for cultivar breeding, market segmentation, growers, and related industries, laying a foundation for further research on food nutrition and human health.

## 1. Introduction

Sweet cherry (*Prunus avium* L.) fruit has become one of the most popular fruits worldwide due to its beautiful skin color, delicious taste, and nutritional value [1]. Approximately 2.6 million tons of it are currently produced globally each year [2]. In China, about 35,730 tons were produced in 2022, with primary contributions from the Shandong, Shaanxi, Liaoning, Sichuan, Hebei, and Shanxi Provinces [3,4]. In Shanxi Province, the planting area for sweet cherry fruit has expanded rapidly over the last three years [4].

The external (in terms of color, size, weight, and physical damage) and internal (in terms of texture, solid-to-acid ratio, and bitterness) quality of sweet cherry fruits affects their consumer acceptance and market price [5,6,7,8,9,10]. Today, the nutrients and bioactive compounds in fruits increasingly influence consumer choices [11,12]. In previous studies, in addition to fruit maturity, temperature, and relative humidity, the cultivar has been identified as an important factor affecting the comprehensive quality of sweet cherry fruits [13,14]. “Tieton”, “Pioneer”, “Sunburst”, and “Huangmi” are the four cultivars mainly grown in Shanxi Province. In this study, we therefore focused on exploring the differences in quality among these four sweet cherry cultivars.

Untargeted metabolomics, a high-throughput technology widely used in fruit quality research, can detect primary and secondary metabolites without bias [13]. For example, Kaleem et al. [14] combined untargeted metabolomics and sensory evaluation to find that the Tianzhen No. 1 rootstock could improve the sensory quality and soluble sugar content of sweet melon fruits. In addition, using untargeted metabolomics, Rocchetti et al. [15] revealed that variety and climatic conditions are the main determinants of functional components (flavonoids, carotenoids) in tomatoes. Thus, using untargeted metabolomics to explore the differences in quality among four sweet cherry cultivars is scientifically supported and feasible.

In this study, we investigated the quality parameters of four sweet cherry fruits (“Tieton”, “Pioneer”, “Sunburst”, and “Huangmi”), including physical damage, bitterness, overall likeability, size, weight, color, texture, and solid-to-acid ratio. Then, we employed untargeted metabolomics to identify differential metabolites (DMEs) among the four cultivars, providing a reference for cultivar breeding, market segmentation, growers, and related industries, and laying a foundation for further research on food nutrition and human health.

## 2. Materials and Methods

### 2.1. Fruit Materials

**Figure 1:**
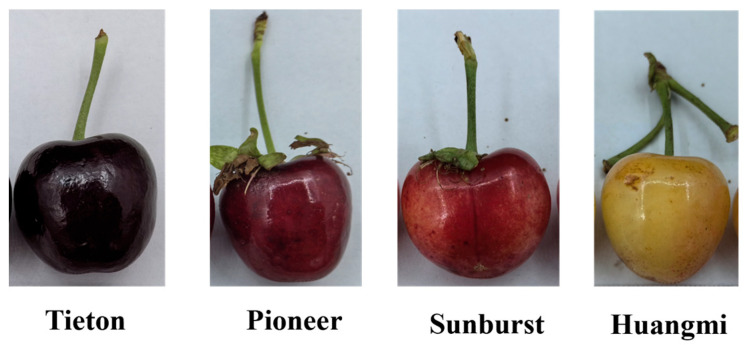
Images of four sweet cherry fruits.

Four sweet cherry fruits (“Tieton”, “Pioneer”, “Sunburst”, and “Huangmi”) at the same maturity level (approximately 90% ripeness) (Figure 1) were harvested in the morning from five trees of each cultivar in an orchard in Shicun, Jiangxian, Yuncheng City, Shanxi Province. The trees, all ten years old, were fertilized and watered in the same manner. After harvest, all the fruits were naturally cooled in a shaded place for about 4 h, and only those with a uniform size, no physical damage, and no pests were selected. The fruits, weighing 2.5 kg, were first placed in a foam box. Then, double layers of tissue paper and two ice packs were laid on the fruits to maintain a low temperature (approximately 10–15 °C). Finally, a lid was placed on the box. The initial relative humidity in the sealed boxes was approximately 55–60%. The fruits were delivered by SF Express to the School of Food Science in Xiaodian District, Taiyuan City, 350 miles away (transport by road, 4 h). Once they arrived, the boxes were opened, and the fruits were checked and stored in a fridge (4 °C and 85–95% relative humidity) as soon as possible. All assessments were conducted the following day. Three boxes of each cultivar, amounting to twelve boxes in total, were used in the experiments.

### 2.2. Reagents and Instruments

All reagents were of analytical-grade purity and were purchased from Aladdin, Macklin, and Kemiou. The following instruments were used: a texture analyzer (TA.XT Plus, Stable Micro Systems Co., Ltd., Godalming, UK), colorimeter (NH310, Shenzhen Sanenshi Technology Co., Ltd., Shenzhen, China), digital display sugar meter (PAL-1, Atago Co., Ltd., Tokyo, Japan), ultra-low-temperature refrigerator (DW-86L58, Jiesheng Co., Ltd., Foshan, China), ultra-high-performance liquid-chromatography system (Vanquish, Thermo Fisher Scientific Co., Ltd., Waltham, MA, USA), mass spectrometer (Q Exactive Focus, Thermo Fisher Scientific Co., Ltd., Waltham, MA, USA) from Personalbio Co., Ltd. (Shanghai, China), and ACQUITY UPLC^®^ HSS T3 column (2.1 × 150 mm, 1.8 µm) (Waters Co., Ltd., Milford, CT, USA).

### 2.3. Physical Damage, Bitterness, Overall Likeability, Size, Weight, and Skin Color

According to the methods reported by Zhang et al. [16] and Brown et al. [17], with some modifications, a trained sensory panel consisting of 15 members investigated overall preference and physical damage. Specifically, 75 representative fruits of each cultivar were selected at first, and then each sensory assessor randomly assessed 5 fruits. Physical damage was rated according to the following criteria: no bruising (0); slight bruising (1); moderate bruising (2); and severe bruising (3). After each sensory assessor smelled and tasted the fruits, they provided a bitterness rating according to the following criteria: no bitterness (0); slight bitterness (1); moderate bitterness (2); severe bitterness (3); and extreme bitterness (4). Finally, overall preference was rated according to the following criteria: extremely dislike (0–4); slightly dislike (5–8); neither dislike nor like (9–12); slightly like (13–16); and extremely like (17–20).

Twenty-one representative fruits were selected from each cultivar, with seven fruits in each group, for a total of 3 groups. The total weight of each group was measured using a weighing instrument and then divided by seven to obtain an average fruit weight. The length, width, and height of each fruit were measured using a vernier caliper. According to the method reported by Yang et al. [18], two locations on the equator of each fruit were selected, and measurements were made using a high-quality computer colorimeter. L* represents brightness; a* represents redness; b* represents yellowness; C represents chroma; and h represents hue.

### 2.4. Texture, Titratable Acidity (TA), Total Soluble Solids (TSS), and Solid-to-Acid Ratio

The firmness, resilience, springiness, gumminess, cohesiveness, and chewiness of individual sweet cherry fruit were determined using the TPA mode of the texture analyzer according to the method reported by Zhang et al. [19], with some modification. The P5 probe was used, with the settings being as follows: 1 mm/s pre-test, test, and post-test speed; 5 mm displacement; and 20 g maximum force. Ten individual fruits of each cultivar were evaluated in this parameter assessment.

Five sweet cherry fruits were stemmed, pitted, wrapped with 4 layers of gauze, and squeezed to yield juice. The TSS of the juice was determined using the digital display sugar meter, while TA was measured according to the method reported by Isildak and Gones [20], with some modification. A blender was used to homogenize the five cherry fruits, and one gram of the samples was then dissolved in 50 mL of distilled water. Two drops of phenolphthalein were added to the mixture, and NaOH (0.1 mol L^−1^) was titrated to a reddish color, with titration ending once the color stopped fading within 30 s. TA was calculated and is expressed as a percentage of malic acid. The solid-to-acid ratio was calculated by dividing TSS by TA. Three replicates of each cultivar were used in the parameter assessments.

### 2.5. Untargeted Metabolomic Analysis of Four Sweet Cherry Fruits

Five sweet cherry fruits were taken as a biological replicate, with three biological replicates of each cultivar collected on day 0, washed with dd H_2_O, frozen with liquid nitrogen for 3 min, and placed in a −80 °C refrigerator for further analysis.

According to the assay reported by Li et al. [21], with slight modification, untargeted metabolomic analysis was conducted. About 20 mg of sweet cherry fruit were first added to 600 µL of methanol containing 2-cholorine-L-phenylalanine, ground to a powder by a mixer mill with a zirconia bead for 1.5 min at 30 Hz. The samples were analyzed by the ultra-high-performance liquid-chromatography system coupled with a mass spectrometer. Samples of 2 μL were injected into the ACQUITY UPLC^®^ HSS T3 column (2.1 × 150 mm, 1.8 µm) using a 17 min linear gradient at a 0.25 mL/min flow rate. For LC-ESI (+)-MS analysis, the mobile phases consisted of 0.1% formic acid in acetonitrile (*v*/*v*) (B) and 0.1% formic acid in water (*v*/*v*) (A). Separation was conducted according to the following gradient: 0~1 min, 2% B; 1~9 min, 2%~50% B; 9~12 min, 50%~98% B; 12~13.5 min, 98% B; 13.5~14 min, 98%~2% B; and 14~20 min, 2% B. Meanwhile, for LC-ESI (−)-MS analysis, the mobile phases consisted of acetonitrile (B2) and ammonium formate (5 mM) (A2). Separation was conducted according to the following gradient: 0~1 min, 2% B2; 1~9 min, 2%~50% B2; 9~12 min, 50%~98% B2; 12~13.5 min, 98% B2; 13.5~14 min, 98%~2% B2; and 14~17 min, 2% B2 [22]. Simultaneous MS1 and MS/MS (full MS-ddMS2 mode, data-dependent MS/MS) acquisition was used, with the following parameters: sheath gas pressure, 30 arb; aux gas flow, 10 arb; spray voltage, 3.50 kV and −2.50 kV for ESI(+) and ESI(−), respectively; capillary temperature, 325 °C; MS1 range, *m*/*z* 100–1000; MS1 resolving power, 70,000 FWHM; number of data-dependent scans per cycle, 3; MS/MS resolving power, 17,500 FWHM; normalized collision energy, 30 eV; and dynamic exclusion time, automatic. The raw data were first converted to mzXML format using MSConvert in the ProteoWizard software package (v3.0.8789) and processed using XCMS in R software (v4.1.2) for feature detection, retention time correction, and alignment. The settings were as follows: bw = 2; ppm = 5; peakwidth = c (5, 30); mzwid = 0.015; mzdiff = 0.01; and method = “centWave”. The metabolites were identified based on mass accuracy (<30 ppm) and MS/MS data, which were matched with HMDB (http://www.hmdb.ca (accessed on 20 May 2024)), massbank (http://www.massbank.jp/ (accessed on 20 May 2024)), LipidMaps (http://www.lipidmaps.org (accessed on 20 May 2024)), mzcloud (https://www.mzcloud.org (accessed on 20 May 2024)), KEGG (http://www.genome.jp/kegg/ (accessed on 20 May 2024)), and the database established by Shanghai Personal Biotechnology Co., Ltd. (Shanghai, China). Quality control-based robust LOESS signal correction was applied for data normalization to correct any systematic bias. After normalization, only ion peaks with relative standard deviations less than 30% in quality control samples were kept, ensuring proper metabolite identification. Each peak area represents the relative content of the corresponding metabolite.

### 2.6. Statistical Analysis

Statistical analysis was performed in IBM SPSS 26 (IBM Corporation, Chicago, IL, USA). All data are expressed as the mean ± standard deviation. For all quality parameters except bitterness, the normal distribution of the data and the homogeneity of variance were first tested. When the data did not fit a normal distribution or the variance was not homogeneous, the Kruskal–Wallis test was used to analyze the data at *p* < 0.05. Otherwise, a one-way analysis of variance (ANOVA) and Duncan’s multiple comparisons were used at *p* < 0.05. For bitterness data, no statistical analysis was conducted because no variability was found within three of the cultivars (“Tieton”, “Pioneer”, and “Sunburst”). Regarding the analysis of the untargeted metabolomic data, Ropls software (v1.6.2) was used to conduct principal component analysis (PCA), partial least-squares discriminant analysis (PLS-DA), and orthogonal partial least-squares discriminant analysis (OPLS-DA). All the models evaluated were tested for overfitting using permutation tests. The descriptive performance of the models was determined using R2X (cumulative) (perfect model: R2X (cum) = 1) and R2Y (cumulative) (perfect model: R2Y (cum) = 1) values, while their predictive performance was measured by Q2 (cumulative) (perfect model: Q2 (cum) = 1) and a permutation test. OPLS-DA enabled the determination of discriminating metabolites using variable importance in projection (VIP). The *P* value, VIP, and fold change (FC) were applied to discover which variables contributed to classification. Finally, *p* < 0.05 and VIP > 1 denoted significant DMEs.

## 3. Results

### 3.1. Comparison of Physical Damage, Bitterness, Overall Likeability, Size, Weight, and Skin Color in Four Sweet Cherry Fruits

**Figure 2:**
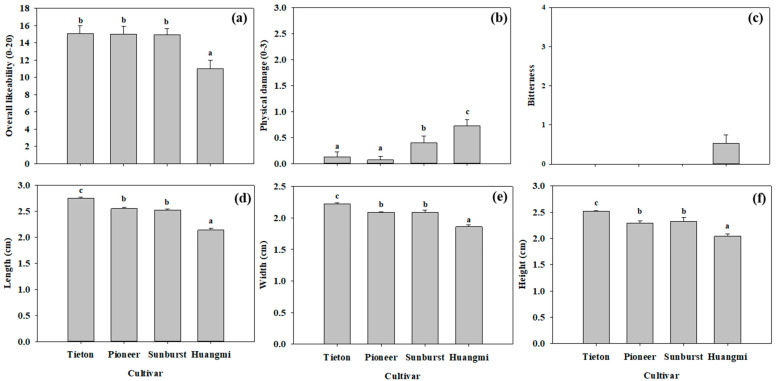
The overall likeability (**a**), physical damage (**b**), bitterness (**c**), length (**d**), width (**e**), and height (**f**) of the four sweet cherry fruits. Seven representative individual fruits were used per replicate, with three replicates for each cultivar measurement. Different letters in each panel indicate significant differences between any two cultivars (*p* < 0.05). Note: In panel (**c**), three cultivars (with a bitterness score of 0) lacked variability, so no statistical comparison was conducted of them.

As shown in Figure 2a, the overall likeability of “Huangmi” fruit was lowest, while that of the other three cultivars (“Tieton”, “Pioneer”, and “Sunburst”) was similar. In Figure 2b, “Huangmi” fruit shows significantly (*p* < 0.05) greater physical damage than “Sunburst”, “Pioneer”, and “Tieton” fruits. Figure 2c shows that “Huangmi” fruit was perceived by people to have a slightly bitter taste, while the other three cultivars were not. As shown in Figure 2d–f, “Tieton” fruit was the largest, with the greatest length, height, and width; “Pioneer” and “Sunburst” fruits were of medium size, with the second-greatest length, height, and width; and “Huangmi” fruit was the smallest, with the lowest length, height, and width values.

**Figure 3:**
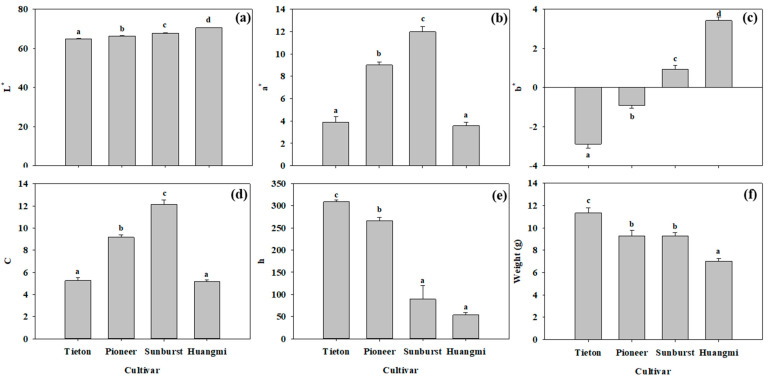
The lightness (L*, (**a**)), redness (a*, (**b**)), yellowness (b*, (**c**)), chroma (C, (**d**)), hue (h, (**e**)), and weight (**f**) of the four sweet cherry fruits. Seven representative individual fruits were used per replicate, with three replicates for each cultivar measurement. Different letters in each panel indicate significant differences between any two cultivars (*p* < 0.05).

Figure 3a reveals that the skin of “Tieton” fruit had the lowest L*, indicating the darkest color, followed by “Pioneer” and “Sunburst”, which ranked second and third, respectively. In contrast, “Huangmi” exhibited the highest L*, corresponding to the brightest skin. As shown in Figure 3b, “Sunburst” displayed the highest a* (indicating the strongest redness), while “Pioneer” ranked second. Both “Tieton” and “Huangmi” exhibited the lowest redness. Figure 3c demonstrates that the b* values of “Tieton” and “Pioneer” were negative, suggesting a bluish tint in their skin. In contrast, “Sunburst” and “Huangmi” had positive b* values, indicating a yellowish color, with “Huangmi” exhibiting greater yellowness than “Sunburst”. Figure 3d illustrates that “Sunburst” had the highest C, representing the most vivid color, followed by “Pioneer”, whereas “Tieton” and “Huangmi” displayed the least vivid color. Figure 3e indicates that the hue values of “Tieton” and “Pioneer” were 309° and 266°, respectively, confirming their reddish appearance. In contrast, “Sunburst” and “Huangmi” were associated with values of 89° and 54°, respectively, reflecting a yellowish color. Based on visual observation, “Tieton” fruit had blackish-purple skin, “Pioneer” fruit had deep-red skin, “Sunburst” fruit had bright-red skin, and “Huangmi” fruit had yellow skin (Figure 1).

### 3.2. Comparison of Texture, TA, TSS, and Solid-to-Acid Ratio of Four Sweet Cherry Fruits

**Figure 4:**
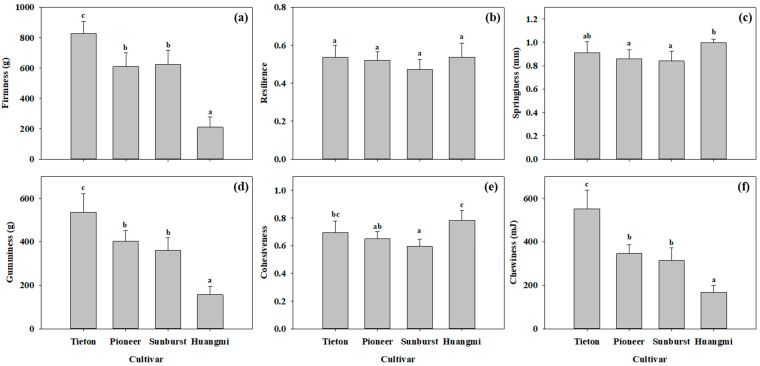
The firmness (**a**), resilience (**b**), springiness (**c**), gumminess (**d**), cohesiveness (**e**), and chewiness (**f**) of the four sweet cherry fruits. Ten representative individual fruits were used for each cultivar measurement. Different letters in each panel indicate significant differences between any two cultivars (*p* < 0.05).

**Figure 5:**
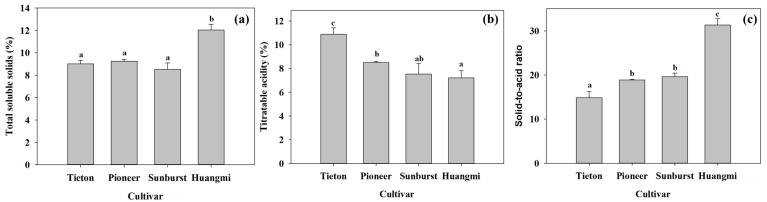
The total soluble solids (TSS, (**a**)), titratable acidity (TA, (**b**)), and solid-to-acid ratio (**c**) of the four sweet cherry fruits. Five individual fruits were used per replicate, with three replicates per cultivar. Different letters in each panel indicate significant differences between any two cultivars (*p* < 0.05).

As shown in Figure 4, “Huangmi” fruit had the lowest firmness, gumminess, and chewiness, and the highest cohesiveness, yielding a soft texture. “Tieton” fruit had the highest firmness, gumminess, and chewiness, and the second-highest cohesiveness, resulting in a hard and crispy texture. “Sunburst” and “Pioneer” had medium firmness, gumminess, and chewiness, with the lowest cohesiveness, resulting in a less hard and crispy texture. Figure 5 shows that “Huangmi” had the highest TSS and solid-to-acid ratio and the lowest TA; “Tieton” had the lowest TSS and solid-to-acid ratio and the highest TA; and “Pioneer” and “Sunburst” had no significant differences in TSS, TA, or the solid-to-acid ratio.

### 3.3. Determination of DMEs Among Four Sweet Cherry Fruits

**Figure 6:**
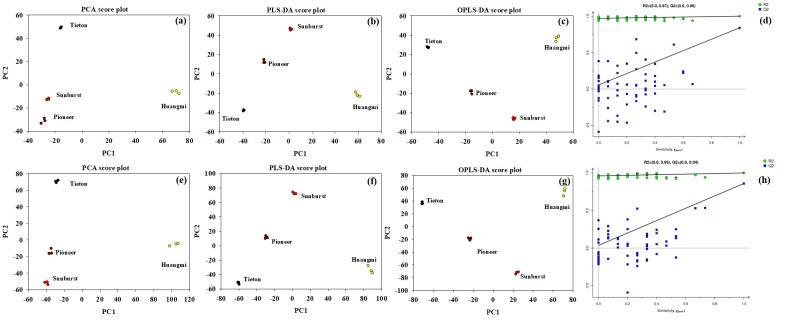
Principal component analysis (PCA, (**a**,**e**)), partial least-squares discriminant analysis (PLS-DA, (**b**,**f**)), orthogonal partial least-squares discriminant analysis (OPLS-DA, (**c**,**g**)), and permutation tests (**d**,**h**) of the OPLS-DA model of differential metabolites (DMEs) among the four sweet cherry fruits in negative and positive modes. Three replicates per cultivar were used.

Through UPLC-MS/MS, 421 metabolites were identified in the flesh of the four sweet cherry cultivars, with 97 DMEs. In the PCA, OPLS, and OPLS-DA models, samples within each cultivar group are clustered together, and the groups are separated (Figure 6a–c,e–g), indicating that these three models could effectively distinguish between the four cultivars. The blue points at the Y-axis intercept are lower than that on the right in the permutation tests of the OPLS-DA model (Figure 6d,h), which demonstrates that the OPLS-DA result is reliable and valid.

**Figure 7:**
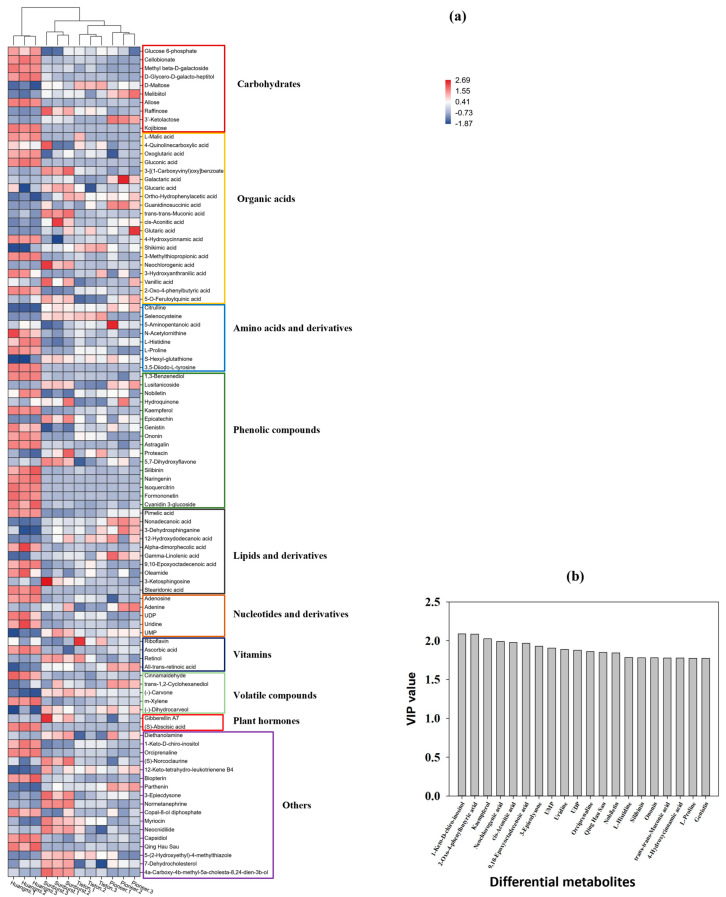
(**a**) Cluster heatmap of significantly differential metabolites (DMEs) among four sweet cherry fruits (“Tieton”, “Pioneer”, “Sunburst”, and “Huangmi”); (**b**) VIP values (top 20) of significantly differential metabolites (DMEs).

As shown in Figure 7a, the DMEs mainly included carbohydrates (10), organic acids (20), amino acids and their derivatives (8), fatty acids and their derivatives (10), phenolic compounds (16), nucleotides and their derivatives (5), vitamins (4), volatile compounds (5), plant hormones (2), and others (17). Relative to “Tieton”, “Pioneer” had the closest genetic relationship, while “Huangmi” was farthest away. Among all the differential carbohydrates, the red cultivars (“Tieton”, “Sunburst”, and “Pioneer”) mainly included glucose 6-phosphate, while the yellow cultivar (“Huangmi”) included glucose 6-phosphate, cellobionate, and methyl β-D-galactoside. However, the content of glucose 6-phosphate in “Huangmi” fruit was 1.5–2.8 times that in the other cultivars. Additionally, “Huangmi” fruit had the highest contents of methyl β-D-galactoside, D-glycero-D-galacto-heptitol, allose, and kojibiose; “Sunburst” fruit had the highest content of raffinose, which was 1.3–2.1 times that in the other cultivars; “Pioneer” fruit had the highest contents of melibiitol and 3′-ketolactose, with the content of the latter being 1.9–4.5 times that in the other cultivars; and “Tieton” fruit showed the highest content of D-maltose, which was 1.1 to 1.5 times that in the other cultivars. In terms of organic acids, the content of malic acid was 3 to 4 orders of magnitude higher than that of other organic acids. Among the four cultivars, the content of malic acid in “Huangmi” was highest, being 2.3 to 4.3 times that in the other cultivars. Additionally, “Huangmi” had the highest content of oxoglutaric acid, gluconic acid, 4-hydrocinnamic acid, 3-methylthiopropionic acid, 3-hydroxyanthranilic acid, and 2-oxo-4-phenylbutyric acid; “Sunburst” had the highest content of 3-[(1-carboxyvinyl)oxy]benzoate, glucaric acid, trans-trans-muconic acid, cis-aconitic acid, and neochlorogenic acid; “Pioneer” had the highest content of galacturonic acid and guanidinosuccinic acid; and “Tieton” had the highest content of shikimic acid, being 1.2 to 1.8 times that in the other three cultivars. In terms of amino acids and their derivatives, the red cultivars (“Tieton”, “Sunburst”, and “Pioneer”) showed higher contents of citrulline than the yellow cultivar (“Huangmi”). In addition, “Huangmi” had the highest content of N-acetylornithine, L-histidine, L-proline, and 3,5-diiodo-L-tyrosine, while “Tieton” had the highest content of selenocysteine. Among the 19 differential phenolic substances, “Huangmi” had the highest content of 11 of them, such as 1,3-benzenediol, kaempferol, and cyanidin-3-glucoside. “Sunburst” had the highest contents of epicatechin and 5,7-didehydroxyflavone, while “Sunburst” and “Pioneer” had the highest content of lusitanicoside. In terms of lipids and their derivatives, “Huangmi” had the highest contents of pimelic acid, α-dimorphecolic acid, 9,10-epoxyoctadecenoic acid, oleamide, and stearidonic acid; “Sunburst” had the highest content of 3-ketosphingosine; and “Pioneer” had the highest contents of nonadecanoic acid and γ-linolenic acid. The contents of these lipid-related DMEs in “Tieton” were generally low. In terms of nucleotides and their derivatives, “Huangmi” had the highest contents of adenosine, uridine, and uridine diphosphate (UDP), while “Sunburst” had the highest content of uridine monophosphate (UMP). In terms of vitamins and plant hormones, “Huangmi” had a high content of ascorbic acid and abscisic acid; “Sunburst” had a high content of retinol and gibberellin A7; “Pioneer” had a high content of all-trans-retinoic acid; and “Tieton” had a high content of riboflavin. In terms of volatile substances, “Huangmi” had high contents of cinnamaldehyde and m-xylene; “Sunburst” had high contents of (−)-carvone and (−)-dihydrocarveol; “Tieton” had a high content of (−)-carvone; and “Pioneer” had high contents of trans-1,2-cyclohexanediol and (−)-dihydrocarveol. Finally, in terms of other substances, “Huangmi” had the highest contents of 1-keto-D-chiro-inositol, biopterin, capsidiol, and Qing Hau Sau; “Sunburst” had the highest contents of diethanolamine, (S)-norcoclaurine, 3-epiecdysone, normetanephrine, myriocin, 5-(2-hydroxyethyl)-4-methylthiazole, 7-dehydrocholesterol, and 4a-carboxy-4b-methyl-5a-cholesta-8,24-dien-3b-ol; and “Pioneer” had the highest content of parthenin. As shown in Figure 7b, the four highest-ranked DMEs in terms of the VIP value were 1-keto-D-chiro-inositol, kaempferol, 2-oxo-4-phenylbutyric acid, and neochlorogenic acid. The content of each differential metabolite differed significantly (*p* < 0.05) among the four cherry cultivars.

## 4. Discussion

Consumers generally prefer sweet cherry fruit with an attractive appearance (such as large and dark red) and delicious taste (such as a high solid-to-acid ratio) [23,24,25]. However, there is no clear evidence so far indicating that fruit texture affects consumer preference for sweet cherries. Our study investigated the quality of four sweet cherry cultivars mainly grown in Shanxi Province and found that “Tieton” fruit was large and had high weight, black-purple skin, a hard and crispy texture, and a low solid-to-acid ratio; “Huangmi” was small in size and had low weight, yellow skin, a soft texture, and a high solid-to-acid ratio; and “Pioneer” and “Sunburst” were medium in terms of their size, weight, and solid-to-acid ratio and had a less-hard and -crispy texture, with dark-red and bright-red skin, respectively. Overall, each sweet cherry cultivar had its advantages. Interestingly, we found that the overall preference for “Huangmi” fruit was lower than that for the other three cultivars. This is possibly because of the greater physical damage to and bitterness of “Huangmi” fruit compared to the other fruits. Physical damage, the first key quality indicator affecting consumers’ overall likeability for a fruit, is closely related to fruit firmness [9,26,27]. The lower a fruit’s firmness is, the more likely it is to suffer physical damage during transportation [28]. In this study, the firmness of the “Huangmi” fruit was indeed found to be lower than that of the other fruits. In general, we propose that pre-harvest treatment or genetic engineering that increases fruit firmness can be an efficient means of improving “Huangmi” fruit quality during transportation.

The primary sugars in sweet cherries include glucose, fructose, and sorbitol, while the main organic acid is malic acid [29,30]. This study found no significant differences in fructose and sorbitol among the four cultivars, though notable differences were detected in glucose 6-phosphate, malic acid, and low-content sugars and organic acids. Glucose 6-phosphate, while not a flavor compound, is a crucial intermediate product of sugar metabolism [31,32]. Higher levels of glucose 6-phosphate were found in “Huangmi” fruit, which may be due to intensified sugar metabolism after the fruits suffered greater physical damage [33]. Conversely, differences in malic acid among the four cultivars may contribute to variations in sourness [34]. Whether low-content sugars and organic acids influence sweetness and sourness, respectively, needs to be studied further. Notably, the “Tieton” cultivar exhibited a shikimic acid content 1.2 to 1.8 times higher than that of other cultivars, which yielded a slightly sour taste according to consumers but did not affect overall likeability. Additionally, a higher content of cellobionate was detected in “Huangmi” fruit than in the other cultivars, which is consistent with the presence of more-fibrous strands in the former.

Sweet cherries are rich in amino acids [35]. This study found that the red-skinned cultivars (“Tieton”, “Pioneer”, and “Sunburst”) had a higher citrulline content than the yellow-skinned cultivar (“Huangmi”), a result that has not been previously reported. Citrulline not only contributes to the sweetness of sweet cherries but also possesses unique physiological activity [36]: it can reduce free radicals, improve vascular function and male sexual function, stimulate muscle protein synthesis, and control cancer development [37,38]. Additionally, we found higher levels of selenocysteine in “Tieton” and “Sunburst” compared to “Pioneer” and “Huangmi”. Selenocysteine is a component of selenoproteins and has antioxidant and anticancer properties [39]. In contrast, higher levels of N-acetylornithine, L-histidine, L-proline, and 3,5-diiodo-L-tyrosine were found in “Huangmi” fruits. L-histidine is an essential amino acid for infants [40], and 5-diiodo-L-tyrosine can be used to treat thyroid diseases [41]. The high accumulation of L-proline may be a stress response to mechanical damage during transportation [42], while that of N-acetylornithine may be due to low enzyme activity in the citrulline synthesis process [43].

Phenolic compounds are typically closely associated with the bitterness, color, and nutritional value of fruits [44]. When tasting “Huangmi” fruits, we detected a mild bitterness that may decrease consumers’ overall likeability. Up to 12 phenolic compounds in “Huangmi” fruit were higher in content than in the other three cultivars. We therefore speculate that the accumulation of phenolic compounds, a response to physical damage, may contribute to its bitterness. A similar proposition was made by Liu et al. with regard to the yellow-skinned “Pengzhoubai” sweet cherry [45]. In addition, anthocyanins and proanthocyanidins jointly determine the color of sweet cherry fruit [45]. This study found that the content of cyanidin-3-glucoside in “Huangmi” was higher than that in the other three red-skinned cultivars, which is consistent with the findings reported by Liu et al. [45].

While lipids in fruits are associated with cell membrane structure and nutritional value, the lipid content in sweet cherry fruits is generally low. This study found that the contents of 12-hydroxydodecanoic acid and 9,10-epoxyoctadecenoic acid were highest in “Huangmi” fruits, possibly due to structural damage to the cell membrane and linoleic acid oxidation following physical damage [46]. However, “Pioneer” fruit had the highest content of γ-linolenic acid and 3-dehydrosphinganine, which to some extent reflects the strong stability of the cell membranes in these fruits [47,48]. In addition, “Pioneer” fruits had the highest content of nonadecanoic acid, which can reduce the risk of diabetes [49].

Nucleotides and their derivatives are closely related to the energy metabolism and nutritional value of fruits. This study found that “Huangmi” fruit had the highest adenosine content, followed by “Tieton”, while “Pioneer” and “Sunburst” had the lowest. Given that adenosine is a product of ATP decomposition [50], we suggest that the energy metabolism of “Huangmi” may be higher than that of “Tieton”, while “Pioneer” and “Sunburst” have the lowest energy metabolism. Moreover, the distribution of UDP and uridine across the four varieties is similar to that of adenosine. Studies have shown that supplementation with uridine and UDP can repair the phospholipids and RNA of neuronal membranes and may be used to treat conditions such as Parkinson’s disease and brain injury [51,52].

Volatile compounds, including esters, ketones, terpenes, aldehydes, and alcohols, are the primary components behind fruit aroma [53,54]. According to our study, cinnamaldehyde, carvone, and dihydrocarveol may be the primary compounds responsible for the characteristic aroma of sweet cherries. In addition, we found that the aromatic compound profiles of the four sweet cherry cultivars differ, indicating that their aromas also vary. These cultivars also exhibit differences in their vitamin content, plant hormones, and bioactive compounds. For example, “Huangmi” had higher levels of ascorbic acid, abscisic acid, 1-keto-D-chiro-inositol, capsidiol, and Qing Hau Sau; “Sunburst” had higher levels of retinol, gibberellin A7, and (S)-norcoclaurine; “Pioneer” had higher levels of all-trans-retinoic acid and parthenin; and “Tieton” had higher levels of riboflavin.

Finally, untargeted metabolomics can effectively distinguish different cultivars. 1-Keto-D-chiro-inositol, kaempferol, 2-oxo-4-phenylbutyric acid, and neochlorogenic acid largely contribute to this discrimination. These metabolites could be candidate markers for distinguishing between other sweet cherry cultivars in the future, though this needs to be verified. This method is different from others that have been published (molecular identification and genetic analysis [55], and image analysis of texture parameters [56]), providing a new approach for growers and related industries.

## 5. Conclusions

“Tieton”, “Pioneer”, “Sunburst”, and “Huangmi” fruits have distinct differences in terms of their appearance, texture, solid-to-acid ratio, nutritional value, and contents of nucleotides and their derivatives as well as volatile and bioactive compounds. In this study, consumer preference for “Huangmi” fruit was lower than that for other fruits, possibly owing to physical damage and fruit bitterness, with the former speculated to be the causative factor for the latter. The resulting accumulation of various phenolic compounds contributes to the bitterness of this fruit. We propose that “Huangmi” fruit quality can be effectively enhanced during transportation through pre-harvest treatment or genetic engineering that increases fruit firmness. Due to its high sugar content, the processing of “Huangmi” fruit into wine or vinegar also represents a favorable option. Meanwhile, “Tieton”, “Pioneer”, and “Sunburst” fruits are suitable for fresh consumption. We also found higher levels of citrulline in the red cultivars (“Tieton”, “Pioneer”, and “Sunburst”) than in the yellow cultivar (“Huangmi”), a result that has not been reported before. In the future, 1-keto-D-chiro-inositol, kaempferol, 2-oxo-4-phenylbutyric acid, and neochlorogenic acid could serve as potential markers for distinguishing between sweet cherry cultivars. This study has only provided preliminary results at present; further research is still needed.

## Data Availability

The original contributions presented in the study are included in the article, further inquiries can be directed to the corresponding authors.
